# Supplementary material for: Toxic effects of sodium dodecyl sulfate on planarian Dugesia japonica
Source: PeerJ. 2023 Jul 10;11:e15660. doi: 10.7717/peerj.15660 (PMC10340106; doi:10.7717/peerj.15660)
Supplement: Table S1 [file peerj-11-15660-s001.docx]

| Primer | Sequence (5’→3’) | GC (%) |
| --- | --- | --- |
| S5 | TGCGCCCTTC | 70 |
| S8 | GTCCACACGG | 70 |
| S10 | CTGCTGGGAC | 70 |
| S15 | GGAGGGTGTT | 60 |
| S17 | AGGGAACGAG | 60 |
| S18 | CCACAGCAGT | 60 |
| S20 | GGACCCTTAC | 60 |
| S64 | CCGCATCTAC | 60 |
| S75 | GACGGATCAG | 60 |
| S78 | TGAGTGGGTG | 60 |
| S80 | ACTTCGCCAC | 60 |
| S83 | GAGCCCTCCA | 70 |
| S84 | AGCGTGTCTG | 60 |
