# Supplementary material for: Toxic effects of sodium dodecyl sulfate on planarian Dugesia japonica
Source: PeerJ. 2023 Jul 10;11:e15660. doi: 10.7717/peerj.15660 (PMC10340106; doi:10.7717/peerj.15660)
Supplement: Table S2 [file peerj-11-15660-s002.docx]

| Primer | Sequences (5’→3’) |
| --- | --- |
| *Dj-Actin* | Forward: ACCAGCAGATTCCATACCCA |
|  | Reverse: TTATGTTACGTTGCCCTCGA |
| *Dj-Caspase3* | Forward: ACGGCAACAGTGAAAGATT |
|  | Reverse: CTAAACAACAGGCAAAGAAA |
| *Dj-PiwiA* | Forward: AGCGGCTCATCATCCTAA |
|  | Reverse: TCCACCACCTGTCCATTT |
| *Dj-PiwiB* | Forward: GCAGTTCATTAGGAGCCA |
|  | Reverse: GGGACAGTCGTAGACAAGG |
| *Dj-PCNA* | Forward: GAATCGTCAAATGAAAGCG |
|  | Reverse: GCAACAGTAATGGTGATAGCC |
| *Dj-CyclinB* | Forward: TGGCAATAATCACTGGTCG |
|  | Reverse: ATGTCTTTGAGCCTTCGTAA |
| *Dj-RAD51* | Forward: ATGTATGGGTCCTTTGCC |
|  | Reverse: CTATTTTATCCACTTTTTGCTC |
