# Supplementary material for: Toxic effects of sodium dodecyl sulfate on planarian Dugesia japonica
Source: PeerJ. 2023 Jul 10;11:e15660. doi: 10.7717/peerj.15660 (PMC10340106; doi:10.7717/peerj.15660)
Supplement: Data S2 — Note: 0.5–1; 0.5–3; and 0.5–5 represent D. japonica exposed to 0.5 mg/L SDS for 1, 3, and 5 days respectively. 1–1; 1–3; and 1–5 represent D. japonica exposed to 1.0 mg/L SDS for 1, 3, and 5 days respectively. [file peerj-11-15660-s004.docx]

Note:

- 0.5-1; 0.5-3; and 0.5-5 represent *D. japonica* exposed to 0.5 mg/L SDS for 1, 3, and 5 days respectively.
- 1-1; 1-3; and 1-5 represent *D. japonica* exposed to 1.0 mg/L SDS for 1, 3, and 5 days respectively.


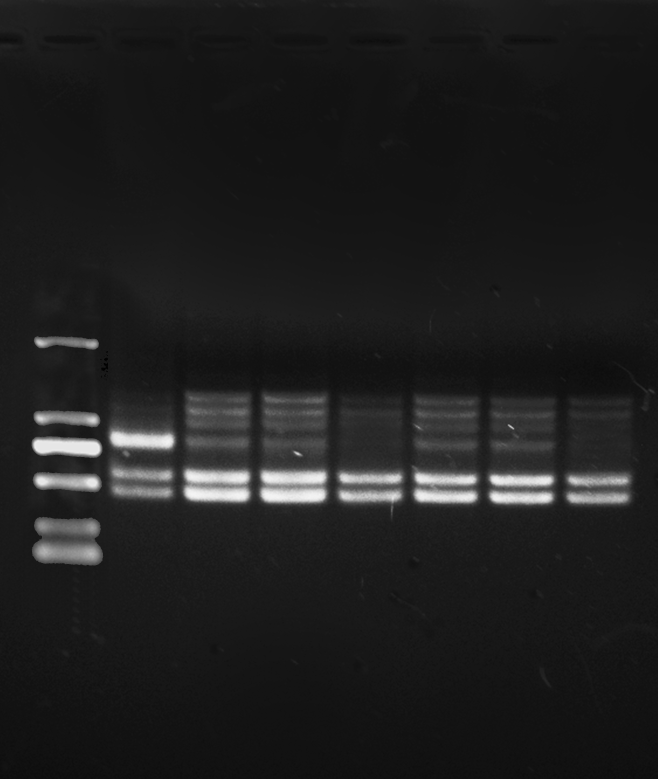


**S5 control 0.5-1 0.5-3 0.5-5 1-1 1-3 1-5**


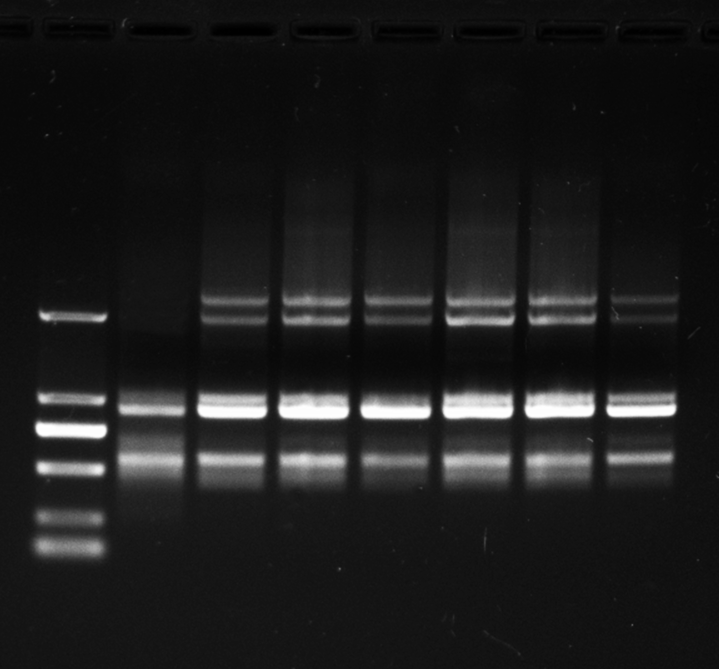


**S8 control 0.5-1 0.5-3 0.5-5 1-1 1-3 1-5**


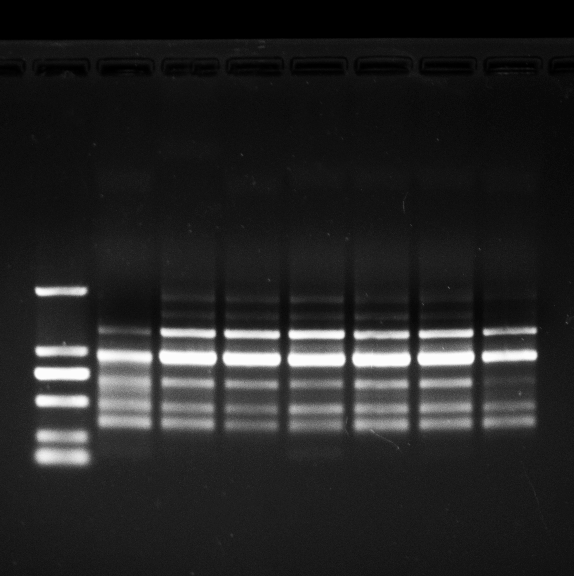


**S10 control 0.5-1 0.5-3 0.5-5 1-1 1-3 1-5**


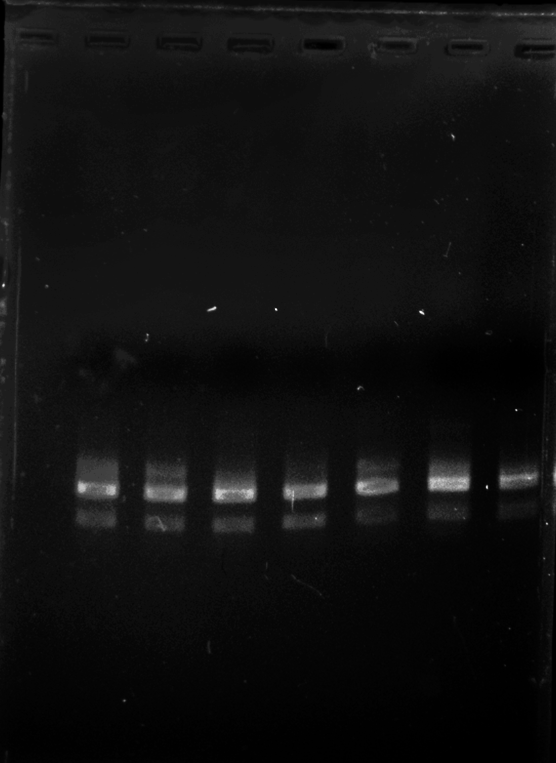


**S15 0.5-1 0.5-3 0.5-5 control 1-1 1-3 1-5**


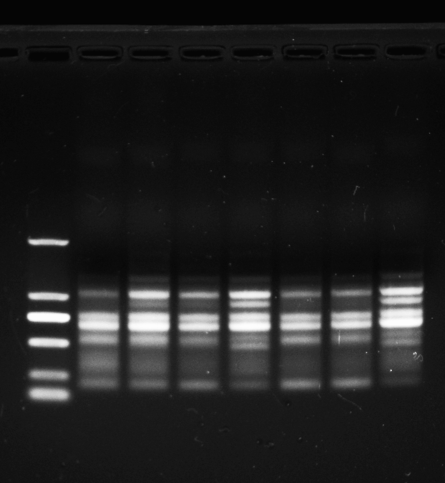


**S17 control 0.5-1 0.5-3 0.5-5 1-1 1-3 1-5**


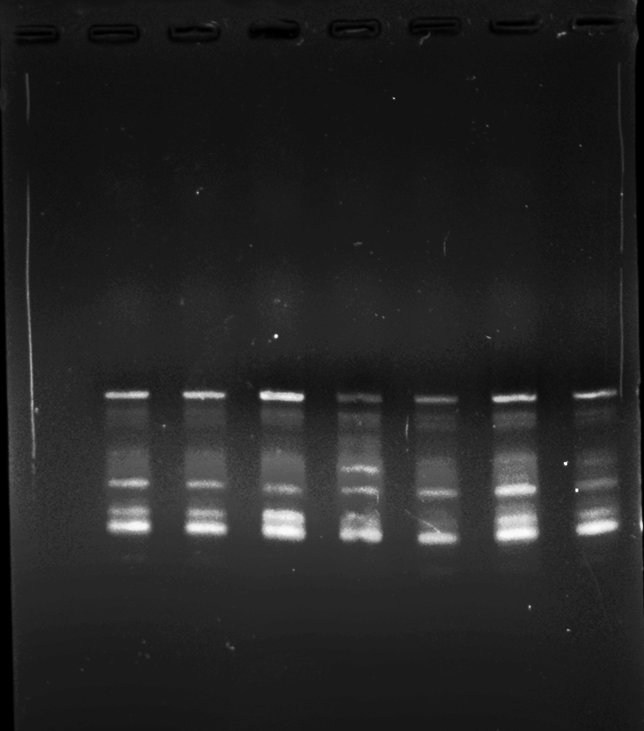


**S18 0.5-1 0.5-3 0.5-5 control 1-1 1-3 1-5**


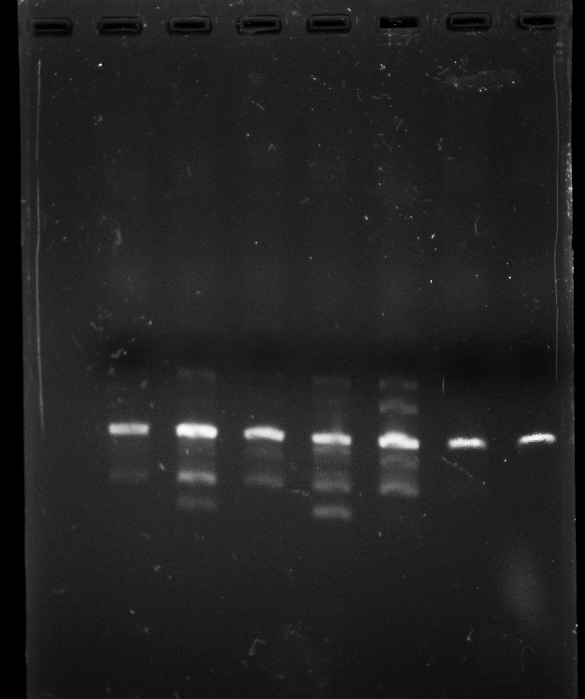


**S20 0.5-1 0.5-3 0.5-5 control 1-1 1-3 1-5**


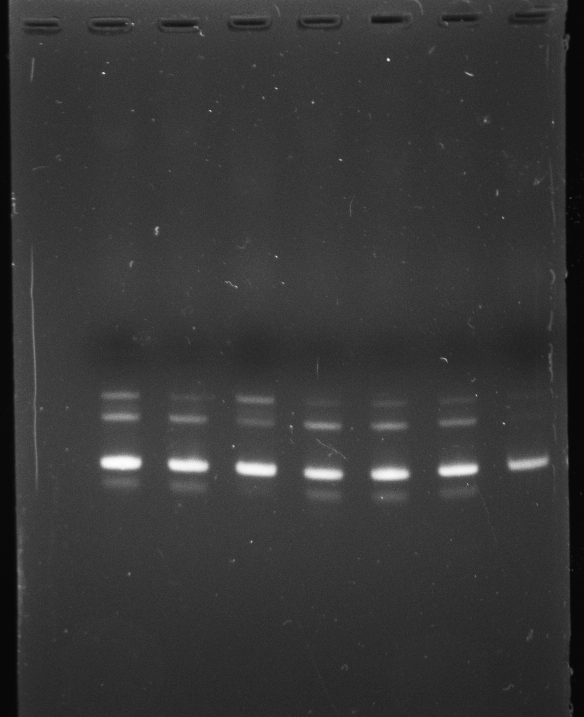


**S64 0.5-1 0.5-3 0.5-5 control 1-1 1-3 1-5**


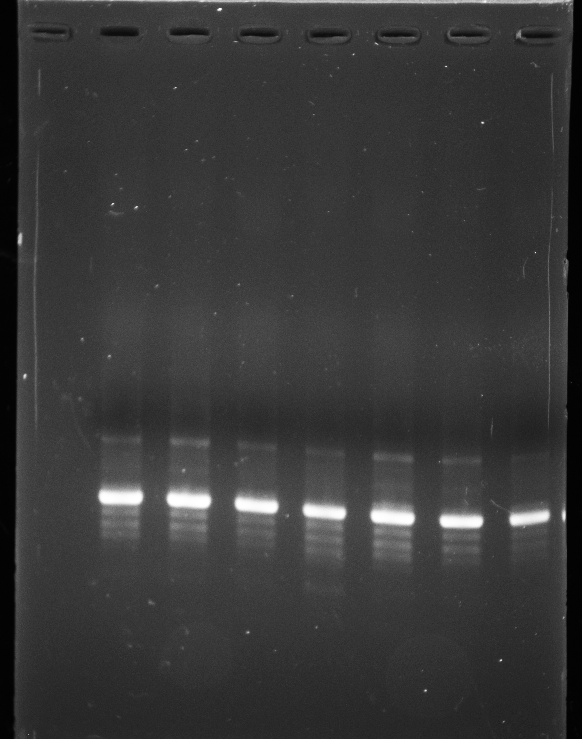


**S75 0.5-1 0.5-3 0.5-5 control 1-1 1-3 1-5**


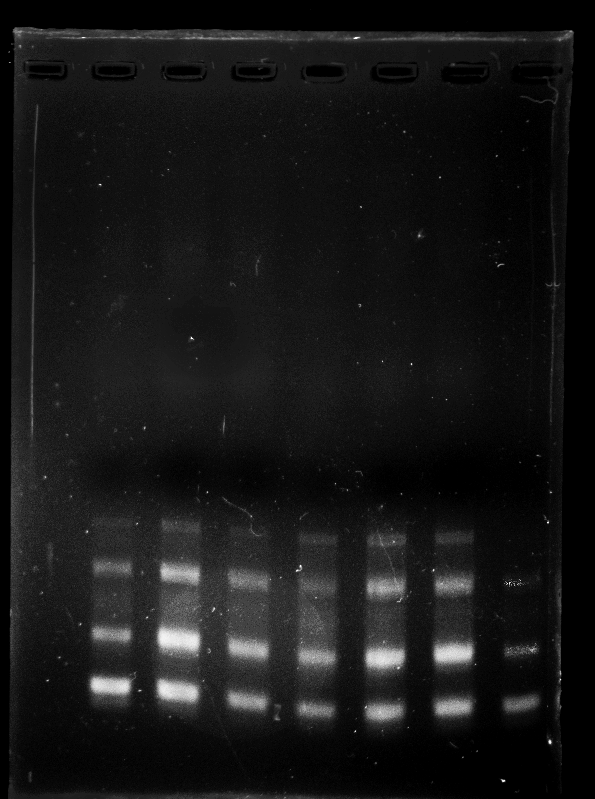


**S78 0.5-1 0.5-3 0.5-5 control 1-1 1-3 1-5**


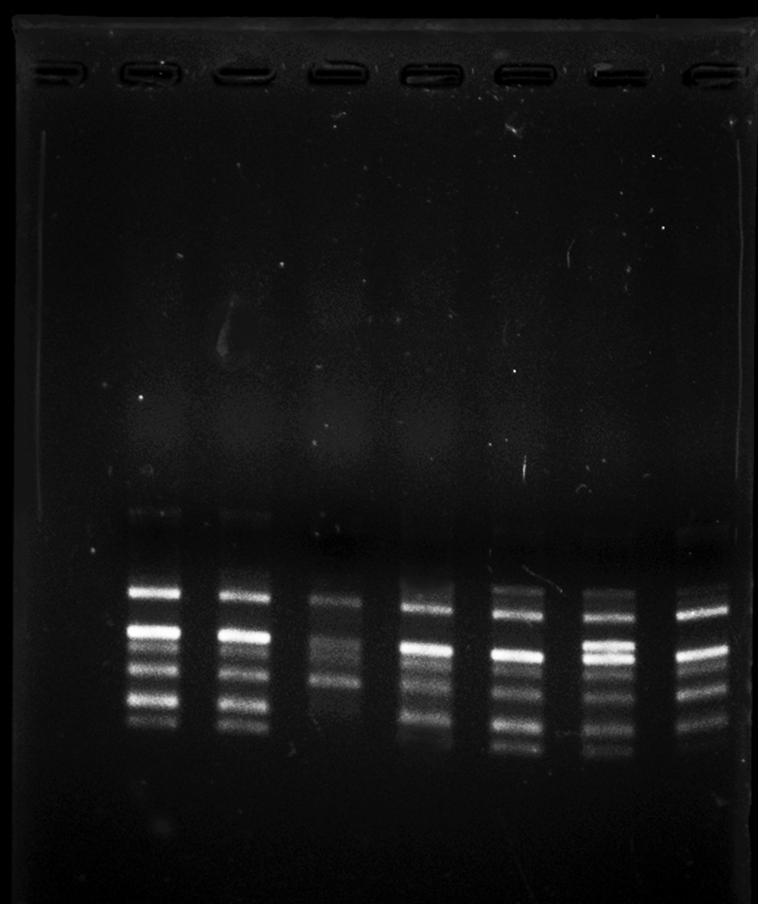


**S80 0.5-1 0.5-3 0.5-5 control 1-1 1-3 1-5**


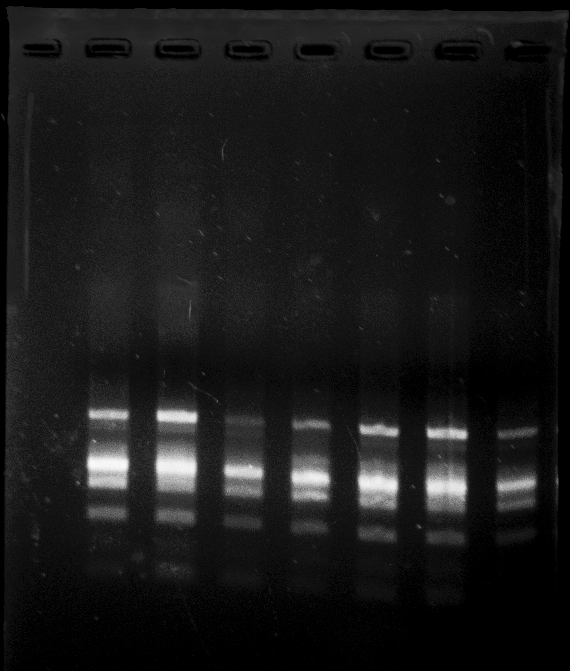


**S83 0.5-1 0.5-3 0.5-5 control 1-1 1-3 1-5**


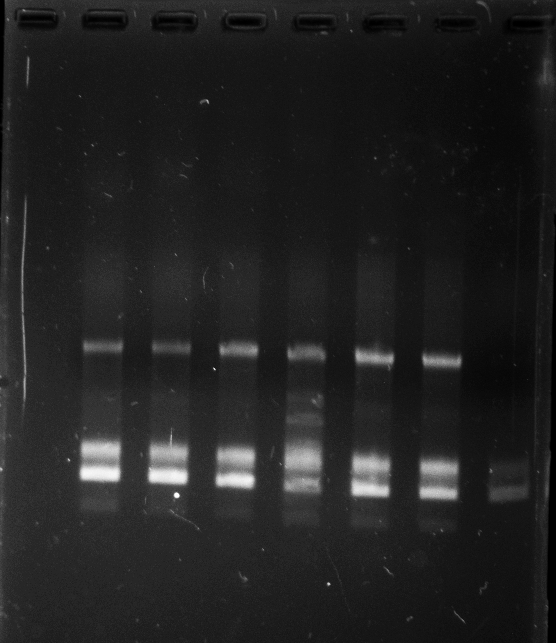


**S84 0.5-1 0.5-3 0.5-5 control 1-1 1-3 1-5**
